# Single-cell conventional pap smear image classification using pre-trained deep neural network architectures

**DOI:** 10.1186/s42490-021-00056-6

**Published:** 2021-06-29

**Authors:** Mohammed Aliy Mohammed, Fetulhak Abdurahman, Yodit Abebe Ayalew

**Affiliations:** 1 School of Biomedical Engineering, Jimma Institute of Technology, Jimma University, Jimma, Ethiopia; 2 Faculty of Electrical and Computer Engineering, Jimma Institute of Technology, Jimma University, Jimma, Ethiopia; 3 Department of Biomedical Engineering, Hawassa Institute of Technology, Hawassa University, Hawassa, Ethiopia

**Keywords:** Deep learning, Image classification, Cervical cancer, Pap smear, CNN

## Abstract

**Background:**

Automating cytology-based cervical cancer screening could alleviate the shortage of skilled pathologists in developing countries. Up until now, computer vision experts have attempted numerous semi and fully automated approaches to address the need. Yet, these days, leveraging the astonishing accuracy and reproducibility of deep neural networks has become common among computer vision experts. In this regard, the purpose of this study is to classify single-cell Pap smear (cytology) images using pre-trained deep convolutional neural network (DCNN) image classifiers. We have fine-tuned the top ten pre-trained DCNN image classifiers and evaluated them using five class single-cell Pap smear images from SIPaKMeD dataset. The pre-trained DCNN image classifiers were selected from Keras Applications based on their top 1% accuracy.

**Results:**

Our experimental result demonstrated that from the selected top-ten pre-trained DCNN image classifiers DenseNet169 outperformed with an average accuracy, precision, recall, and F1-score of 0.990, 0.974, 0.974, and 0.974, respectively. Moreover, it dashed the benchmark accuracy proposed by the creators of the dataset with 3.70%.

**Conclusions:**

Even though the size of DenseNet169 is small compared to the experimented pre-trained DCNN image classifiers, yet, it is not suitable for mobile or edge devices. Further experimentation with mobile or small-size DCNN image classifiers is required to extend the applicability of the models in real-world demands. In addition, since all experiments used the SIPaKMeD dataset, additional experiments will be needed using new datasets to enhance the generalizability of the models.

## Background

Cervical cancer is a women-specific sexually transmitted infectious disease caused by, mainly, high-risk human papillomavirus (HPV). Worldwide, an estimated 570,000 cases and 311,000 deaths were registered in 2018 only. Among these numbers, about 85% of them are from developing countries [1].

Considering the prevalence of the disease, international organizations such as the World Health Organization (WHO) start to set new initiatives to eliminate it from the public health burden. WHO’s new strategy emphasized on the elimination of cervical cancer from public health problems before the year 2030, mainly, focusing on three pillars (prevention, screening and treatment/ management) in a comprehensive approach. In the strategy, it is clearly stated that to reach the stage of cervical cancer elimination, every country must give 90% coverage of HPV vaccine for girls of 15 years of age, perform 70% high-performance cervical cancer test (screening) for females between ages of 35 and 45, treat 90% of precancerous lesions and 90% management of invasive cancer patients [2].

In the past few decades, high-income countries have implemented population-wide screening programs and showed a significant reduction in mortality and morbidity caused by cervical cancer [3, 4]. The experience could be a good model to be further extended in low- and middle-income countries. However, the lack of basic resources such as skilled health personnel and screening tools have been posing a major challenge [5, 6].

The latest WHO guideline regarding cervical cancer screening recommends three main techniques: high-risk HPV type testing using polymerase chain reaction (PCR), visual inspection with acetic acid (VIA), and cervical cytology [7]. Among the three, cervical cytology is the most common and the orthodox way of screening. It has been considered as the standard technique valuing its contribution to the reduction of incidence and mortality rate in many high-income countries worldwide [5]. The popular and well-developed techniques for cervical cytology are conventional Papanicolaou smears (CPS) and liquid-based cytology (LBC). The results of comparative studies focusing on the quality of CPS and LBC samples concluded that LBC is better than CPS [8, 9]. However, considering the economic burden, LBC is more common in high-income countries whereas CPS is more preferable in low- and middle-income countries [8].

Even though cytology techniques are effective in the reduction of morbidity and mortality, they suffered from the following main drawbacks: their sensitivity is less optimal, the interpretation of results mainly depends on the morphological characteristics of cytoplasm and nucleus of the cell which requires a highly skilled cytotechnologist. Moreover, analyzing a single sample takes considerably long time and is labor-intensive.

In order to bridge the aforementioned gaps of manual cervical cytology screening, computer vision experts have been developing alternative computer aided analysis tools, especially for CPS based analysis. Automated computer aided tools should work on par with medical experts in order to deploy them in real world environments. The recent advancement of the computer vision field has benefited from deep learning algorithms and has shown very promising results for medical image analysis. Researchers have developed systems that either classify single-cell CPS images or detect abnormal cells from full-slide CPS images. A detailed and extended review is found in [10].

**Fig. 1:**
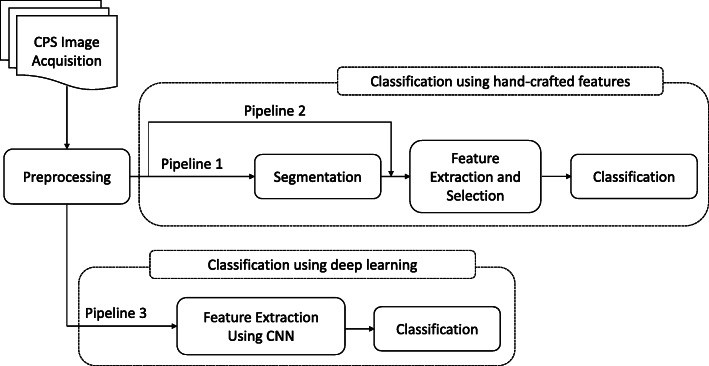
Common pipelines to classify CPS images

In literature, three single-cell CPS image analysis pipelines have been proposed as illustrated in Fig. 1. The traditional techniques follow either pipeline 1 or pipeline 2 or both combined, which are based on handcrafted features generated from either segmented regions of the CPS images or directly from the preprocessed CPS images. The main difference between the two pipelines is the requirement of the segmentation stage. For instance, if the required feature vectors are attributes of the morphology or shape of an object such as area, perimeter, thinness ratio, and eccentricity, first, the cytoplasm or the nucleus of the cells need to be segmented from the rest of the image content. On the other hand, if the required features do not require descriptors of segmented objects such as chromatin and texture, the segmentation stage will be skipped as it is depicted in the pipeline 2. In other words, the feature vectors will be directly calculated from the pre-processed CPS images. Features calculated using the two pipelines commonly known as hand-crafted features. Hand-crafted features give a privilege to the computer vision expert to select and supervise the extracted feature vectors. Sometimes dimensionality reduction schemes pick the right subset from a bucket of large feature vectors. Previous research works had been presented using such traditional single-cell CPS image analysis techniques [11–18]. The other technique (pipeline 3) takes the benefit of deep convolutional neural networks (DCNNs) to learn complex features directly from the labelled raw or preprocessed CPS images. The main advantage of these deep DCNNs is their ability to extract feature vectors without the intervention of domain experts. Previous studies that used DCNNs for single-cell CPS analysis are presented in [19–24]. In this study, we investigated the applicability and performance of transfer learning for single-cell CPS image analysis using pre-trained DCNNs.

Plissiti, M. E et al. [17] produced a new benchmark CPS dataset in 2018 named SIPaKMeD which is used by researchers for both traditional and deep learning based CPS image analyses. In [17] they have used VGG-19 architecture for classification of the SIPaKMeD dataset into 5-classes. They have also used SVM at the last convolution layer and fully connected layer to classify pre-activated features extracted using the VGG-19 model. For the deep learning based classification benchmark, they achieved an average accuracy of 95.35, 93.35 and 94%, respectively.

To the best of the authors’ knowledge there is no research that bases [17] as a benchmark and SIPaKMeD as dataset. In this study, we contributed by exploring ten best performing DCNN image classifiers which are selected based on their top-1 accuracy on ImageNet classification challenge. We have conducted detailed transfer learning experiments using the selected pre-trained DCNN image classifiers and performed a comprehensive comparative analysis with the benchmark research. In addition, we have applied preprocessing algorithms to boost the performances of the classification schemes. As a limitation, due to lack of similar cytology datasets, we haven’t evaluated the proposed schemes on other datasets. This probably affect the generalization ability of the classification models when they encounter single-cell CPS images collected from a different setting to the SIPaKMeD dataset.

### Experiment and results

**Fig. 2:**
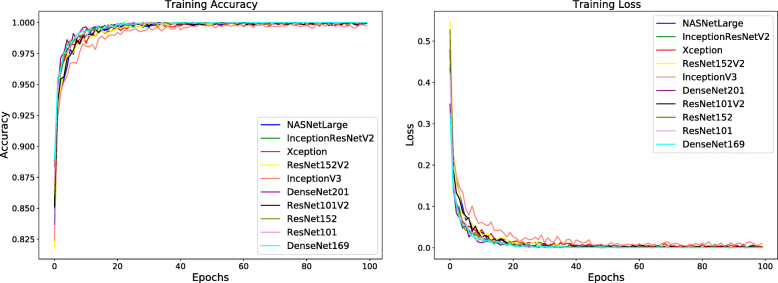
Training accuracy (left) and training loss (right) of the proposed classification models

**Fig. 3:**
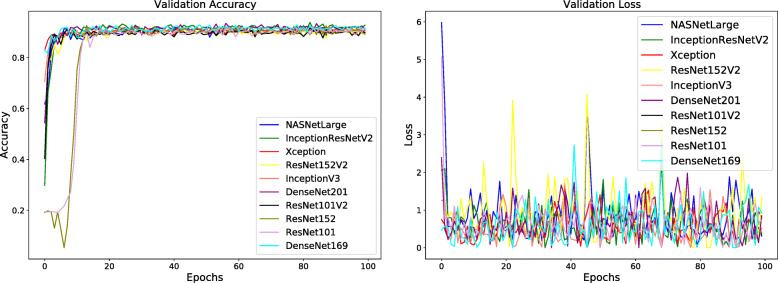
Validation accuracy (left) and validation loss (right) of the proposed classification models

To maintain a fair comparison, all the training hyperparameters were kept identical in all experiments. As illustrated in Figs. 2 and 3 the networks were trained for 100 epochs using a categorical cross-entropy loss, a batch size of 32 and adagrad optimizer. We have trained all the models with an initial learning rate of 0.001 which changes its value by a factor of 0.5 if there is no increment in the validation accuracy over 10 consecutive epochs until it reaches a value of 0.00001.

After training, we evaluated the classification models using the test dataset and their evaluation results are summarized in Table 1.

**Table 1:** Individual and average accuracies, precisions, recalls and F1-scores of the proposed classification models evaluated using test dataset

		Accuracy	Precision	Recall	F1-score			Accuracy	Precision	Recall	F1-score
**NASNetLarge**	**DC**	0.992	0.980	0.980	0.980	**InceptionResNetV2**	**DC**	0.992	0.962	1.000	0.980
	**KC**	0.964	0.894	0.930	0.912		**KC**	0.958	0.899	0.890	0.894
	**MC**	0.970	0.913	0.940	0.926		**MC**	0.968	0.904	0.940	0.922
	**PC**	0.994	0.990	0.980	0.985		**PC**	0.988	0.980	0.960	0.970
	**SIC**	0.988	1.000	0.940	0.969		**SIC**	0.990	1.000	0.950	0.974
	**Average**	0.982	0.955	0.954	0.954		**Average**	0.979	0.949	0.948	0.948
**Xception**	**DC**	0.990	0.961	0.990	0.975	**ResNet152V2**	**DC**	0.986	0.952	0.980	0.966
	**KC**	0.968	0.920	0.920	0.920		**KC**	0.958	0.891	0.900	0.896
	**MC**	0.974	0.922	0.950	0.936		**MC**	0.968	0.904	0.940	0.922
	**PC**	0.986	0.989	0.940	0.964		**PC**	0.992	0.990	0.970	0.980
	**SIC**	0.998	1.000	0.990	0.995		**SIC**	0.988	1.000	0.940	0.969
	**Average**	0.983	0.959	0.958	0.958		**Average**	0.978	0.947	0.946	0.946
**InceptionV3**	**DC**	0.988	0.943	1.000	0.971	**DenseNet201**	**DC**	0.988	0.961	0.980	0.970
	**KC**	0.964	0.936	0.880	0.907		**KC**	0.964	0.918	0.900	0.909
	**MC**	0.966	0.888	0.950	0.918		**MC**	0.978	0.941	0.950	0.945
	**PC**	0.984	0.979	0.940	0.959		**PC**	1.000	1.000	1.000	1.000
	**SIC**	0.994	1.000	0.970	0.985		**SIC**	0.998	1.000	0.990	0.995
	**Average**	0.979	0.949	0.948	0.948		**Average**	0.986	0.964	0.964	0.964
**ResNet101V2**	**DC**	0.986	0.951	0.980	0.966	**ResNet152**	**DC**	0.992	0.971	0.990	0.980
	**KC**	0.964	0.918	0.900	0.909		**KC**	0.968	0.912	0.930	0.921
	**MC**	0.962	0.893	0.920	0.906		**MC**	0.974	0.939	0.930	0.935
	**PC**	0.994	0.980	0.990	0.985		**PC**	0.996	1.000	0.990	0.995
	**SIC**	0.990	1.000	0.950	0.974		**SIC**	0.992	1.000	0.980	0.990
	**Average**	0.979	0.949	0.948	0.948		**Average**	0.986	0.964	0.964	0.964
**ResNet101**	**DC**	0.992	0.980	0.980	0.980	**DenseNet169**	**DC**	0.998	1.000	0.990	0.995
	**KC**	0.962	0.909	0.900	0.905		**KC**	0.974	0.922	0.950	0.936
	**MC**	0.972	0.913	0.950	0.931		**MC**	0.978	0.941	0.950	0.945
	**PC**	0.998	1.000	0.990	0.995		**PC**	0.998	1.000	0.990	0.995
	**SIC**	0.996	1.000	0.980	0.990		**SIC**	0.998	1.000	0.990	0.995
	**Average**	0.984	0.961	0.960	0.960		**Average**	**0.990**	**0.974**	**0.974**	**0.974**

## Discussion

As can be inferred from Table 1, DenseNet169 outperforms all the other classification models in all evaluation metrics. Its normalized average accuracy, precision, recall and F1-score values are 0.990, 0.974, 0.974 and 0.974, respectively. Across all experiments, Koilocytotic cells are more challenging to classify, i.e. their true positive value is the least compared to other classes. Similar reporting can be found in the benchmark manuscript [17]. The second most challenging class type is the metaplastic cells.

**Fig. 4:**
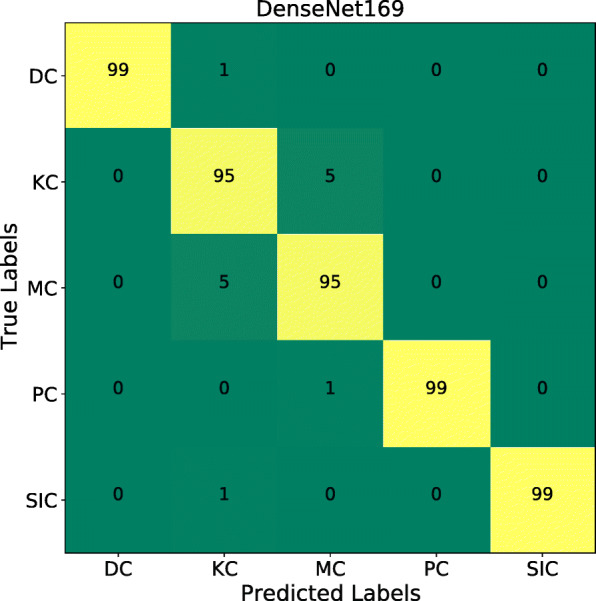
Confusion matrix for classification result on test dataset using DenseNet169

When we further inspected the aforementioned cell types, we found out that most of the false negatives of Koilocytotic cells were incorrectly classified as metaplastic and most of the metaplastic cells were incorrectly classified as Koilocytotic cells as shown in the confusion matrix of DenseNet169 in Fig. 4. This experimental result tells us the need to increase the data variation between the two classes.

During our experimental analysis we have also inspected the size of the weight files of our proposed pre-trained classification models. DenseNet169 has the smallest weight size (Table 2 shows the size of the original weight file) which is also our best performing model. However, still, the large weight file size makes it unsuitable to deploy to mobile or edge devices. As a future research direction we want to analyze how to develop classification models having high accuracy with minimal memory and computation consumption.

**Table 2:** Proposed pre-trained classification models weight size and their top-1 accuracy performance on the ImageNet’s validation dataset

Model	Size	Top-1 Accuracy
NASNetLarge	343 MB	0.825
InceptionResNetV2	215 MB	0.803
Xception	88 MB	0.790
ResNet152V2	232 MB	0.780
InceptionV3	92 MB	0.779
DenseNet201	80 MB	0.773
ResNet101V2	171 MB	0.772
ResNet152	232 MB	0.766
ResNet101	171 MB	0.764
DenseNet169	57 MB	0.762

Finally, we compared our findings with the work done by [17] with a similar dataset as our benchmark. In their work, they presented an average accuracy of 95.35 ± 0.42% using VGG19 as a feature extractor and softmax as a classifier. In this research, we achieved a normalized average accuracy of 0.990 which is significantly better than the benchmark work.

## Conclusion

In this paper, we presented a single-cell CPS image classification model using pre-trained deep convolutional neural network algorithms. The pre-trained models were selected based on their top-1 accuracy on ImageNet classification dataset. We have done detailed experiments on the selected pre-trained DCNN image classification models by fine-tuning and selecting network hyperparameters to achieve best classification accuracy. All the pre-trained DCNN image classifiers were fine-tuned to suit SIPaKMeD dataset by changing the final fully connected and output layer of the classifiers. From the selected 10 pre-trained DCNN image classifiers, DenseNet169 outperformed the other architectures and achieved state-of-the-art performance compared to the benchmark result generated by the SIPaKMeD dataset creators. Using DenseNet169 a normalized average accuracy of 0.990 was achieved which is greater than the benchmark by approximately 3.70%. In the future, further experimentation with small size and mobile DCNN image classifiers is required to make the size of model weights suitable for mobile and edge devices. Alongside small size image classifiers, recent optimizers that performed well in other domains such as Chimp optimization algorithm (ChOA) [25] need to be explored to achieve high performance. In addition, the proposed pre-trained classification models should be tested in datasets from different data acquisition environments in order to increase their generalization capability of the models in real-time clinical settings.

## Materials and methods

The general flow diagram of the proposed method is illustrated in Fig. 6. Our proposed method consists of data acquisition and pre-processing, feature extraction using different DCNN architectures and finally classifying the input image of Pap smear into pre-defined five classes. Each of the components in our method are described in detail on the following subsections.

### Dataset

**Fig. 5:**
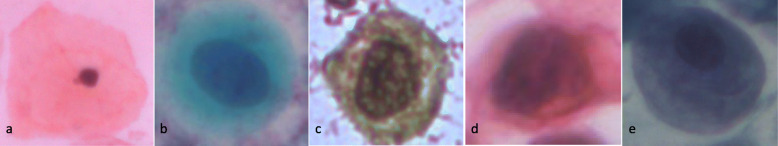
Sample images from the SIPaKMeD dataset: superficial-intermediate (a), parabasal (b), koilocytotic (c), metaplastic (d) and dyskeratotic (e) cells

In this study, a recently introduced publicly available dataset named SIPaKMeD was used [17]. The dataset contains a total number of 4049 single-cell images that were manually cropped from 966 full-slide Pap smear images. The cells were grouped based on their abnormality and benign level into 5 classes - superficial-intermediate cells (SIC), parabasal cells (PC), koilocytotic cells (KC), dyskeratotic cells (DC) and metaplastic cells (MC). The first two are normal, the second two are abnormal and the last one is benign. The distribution of images across the single-cell image classes is seemingly uniform - 831, 787, 825, 793 and 813, respectively. Figure 5 shows representative images of the five classes.

We randomly partitioned the dataset into training, testing and validation sets. We have used 12% of the dataset for testing and the remaining 88% is used as training and validation dataset with percentiles of 80 and 20, respectively.

We have pre-processed the dataset before feeding into the classification network. We have performed image resizing, image normalization, affine transformations, and class balancing. All images (training, validation and test) were resized to 128x128x3 to reduce the computation overhead which is experimentally selected with optimal performance. Image normalization was done to keep the dynamic range of pixel intensities of the images between 0 and 1. Affine transformations were done on the training and validation sets to increase intra class variation during training. The selected affine transformations were flipping (both horizontally and vertically) and rotation (ranged between − 45^0^ and 45^0^). Even though the cross-class distribution is considerably uniform (the ratio between the classes with the smallest to the largest number of images is approximately 0.95), we applied class weight balancing on the training and validation dataset using Eq. 1. At the time of training, the distribution of the classes for individual batches turned out to be 0.97, 1.03, 0.98, 1.02 and 1.00 for SIC, PC, KC, MC and DC, respectively.

$$ {w}_j=\frac{S}{n\ast {s}_j} $$--- Eq. 1.

Where, *w*_*j*_ stands for the weight of the class *j*, *S* for the total number of samples, *n* for the number classes and *s*_*j*_ for the samples in the class *j*.

### Proposed approach

In this study, as illustrated in Table 2, we selected top 10 popular pre-trained DCNN image classifiers from Keras applications [26] based on their top-1 accuracy tested on ImageNet validation dataset. Top-1 accuracy refers to the normalized performance of a model to predict exactly the expected answer. For example, the probability of NASNetLarge to predict exactly the first answer is 0.823 out of a unit scale. The selected modes were trained on ImageNet [27] - a dataset of 1000 classes of natural images.

Recent advancements in DCNN has remarkably enhanced the performance of image classification algorithms. However, their use for medical image classification is challenging since training deep models need an enormous amount of data. Transfer learning has become one of the most popular techniques for enhancing the performance of machine learning models which is used to adapt knowledge learned in source data to target dataset. The approach will be important for medical image classification where we cannot find enough dataset to train from scratch.

**Fig. 6:**
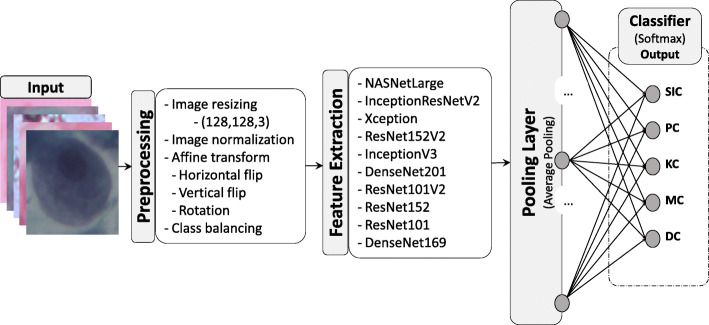
The general pipeline of the research project: image acquisition, pre-processing, feature extraction and classification

In this study, considering the SIPaKMeD dataset size which is small we have used pre-trained models on the ImageNet dataset and fine-tuned them using the target SIPaKMeD dataset. In other words, the weights of the feature extraction base were re-trained again using the CSP dataset to populate it with new weights and the output layer was changed from 1000 classes down to 5 classes. To converge the output of the feature extraction base from 4D tensor to a 2D tensor an average pooling layer was introduced. At the end, fully connected links were created between the pooling layer and the output dense layer as indicated in Fig. 6.

In our experimental design, we took the pre-trained weight files of the selected classification models and fine-tuned them using SIPaKMeD dataset. We have changed the final fully connected heads in all the models with one fully connected layer with 512 neurons. In all the models we replace the final classification layer which is 1000 classes in the pre-trained models into 5 classes. We have also applied affine transformation as a data augmentation technique to increase the size of our limited data which helps to prevent the class imbalance problems and model overfitting. All the experiments were performed using Google’s free cloud platform, Kaggle, with NIvida Tx1008 GPU and 12 GB of RAM.

### Evaluation metrics

We evaluated the performance of the classification models using four objective evaluation metrics including accuracy, precision, recall and f1-score. The metrics base their mathematical foundation on the true positive (TP), true negative (TN), false negative (FN) and false positive (FP) values of the models’ prediction. A comprehensive summary of the metrics is found in [28] and their mathematical formulation as follows.

$$ Accuracy=\frac{TP+ TN}{TP+ TN+ FN+ FP} $$

$$ Precision=\frac{TP}{TP+ FP} $$

$$ Recall=\frac{TP}{TP+ FN} $$

$$ F1- Score=2\ast \left(\frac{Precision\ast Recall}{Precision+ Recall}\right) $$

## Data Availability

The minimal dataset used for this study was extracted from the original SIPaKMeD dataset. Link to the original dataset - https://www.cs.uoi.gr/~marina/sipakmed.html Link to the minimal dataset - https://www.kaggle.com/mohaliy2016/papsinglecell/

## Abbreviations

CNN: Convolutional Neural Network
CPS: Conventional Papanicolaou Smears
DCNN: Deep Conventional Neural Network
DC: Dyskeratotic Cells
FN: False Negative
FP: False Positive
KC: Koilocytotic Cells
LBC: Liquid Based Cytology
MC: Metaplastic Cells
PC: Parabasal Cells
SIC: Superficial-Intermediate Cells
TN: True Negative
TP: True Positive
SVM: Support Vector Machine
WHO: World Health Organization
VIA: Visual Inspection with Acetic acid
